# Advances of regional nerve blocks for postoperative analgesia in laparoscopic gynecological surgery: precision, innovation, and future directions

**DOI:** 10.3389/fmed.2026.1731301

**Published:** 2026-03-03

**Authors:** Zhenhong Zhou, Maolin Zhong, Shihong Li

**Affiliations:** 1 The First Clinical Medical College of Gannan Medical University, Ganzhou, China; 2 Department of Anesthesiology, The First Affiliated Hospital of Gannan Medical University, Ganzhou, China; 3 Ganzhou Key Laboratory of Anesthesiology, The First Affiliated Hospital of Gannan Medical University, Ganzhou, China

**Keywords:** enhanced recovery after surgery, laparoscopic gynecological surgery, postoperative analgesia, regional nerve block, ultrasound guidance

## Abstract

Laparoscopic gynecological surgery, despite its minimally invasive nature, is frequently associated with significant postoperative pain, encompassing somatic, visceral, and referred components. This pain poses challenges to patient recovery and increases opioid consumption, highlighting the need for effective, opioid-sparing strategies within Enhanced Recovery After Surgery (ERAS) pathways. This narrative review explores the evolution of regional nerve blocks as a cornerstone of postoperative pain management in this surgical context. It traces the progression from early, nonspecific techniques such as local infiltration to the modern era of precise, ultrasound-guided fascial plane blocks. The evidence supporting major truncal blocks including the Transversus Abdominis Plane Block (TAPB), Quadratus Lumborum Block (QLB), and Erector Spinae Plane Block (ESPB) is critically examined, demonstrating a shift from primarily somatic analgesia to techniques that also address visceral pain. Approaches to optimizing block efficacy and duration, including the use of pharmacological adjuvants (e.g., dexamethasone, dexmedetomidine) and dose-optimization strategies, are discussed. The synthesis of current evidence underscores the role of regional nerve blocks as a foundational component of modern multimodal analgesia, essential for facilitating early recovery and improving patient outcomes. Looking ahead, the field is moving toward personalized analgesia, where block selection is tailored to the specific surgical “pain fingerprint” and individual patient needs, guided by ongoing advancements in technology.

## 1 Introduction

The advent of laparoscopic surgery in the late 20th century marked a paradigm shift in modern surgical practice. Since the introduction of laparoscopic hysterectomy in 1989, this approach has demonstrated multiple benefits, including reduced pain, shorter hospital stays, superior cosmetic outcomes, and faster recovery (1). As a result, laparoscopic techniques are now considered the standard of care for a wide range of benign and malignant gynecological conditions.

Despite being classified as minimally invasive, laparoscopic procedures are frequently associated with significant postoperative pain. Ekstein et al. (2) highlighted that these procedures often require substantial analgesia during the immediate postoperative period. Among gynecological surgeries, total laparoscopic hysterectomy is associated with a particularly complex and prolonged pain profile (3, 4), underscoring the pressing need for targeted and effective pain management strategies.

Historically, postoperative pain management has relied heavily on opioids. While effective, opioids are associated with numerous adverse effects (5). The ongoing opioid crisis has prompted a shift toward opioid-sparing regimens, in alignment with Enhanced Recovery After Surgery (ERAS) protocols. These protocols prioritize multimodal analgesia to achieve superior pain control while minimizing opioid consumption (6–9).

Within the context of ERAS, regional nerve blocks have emerged as a cornerstone for optimizing postoperative pain control in laparoscopic gynecological surgery. This review critically evaluates the evidence supporting major abdominal wall and truncal blocks, including the Transversus Abdominis Plane Block (TAPB), Quadratus Lumborum Block (QLB), and Erector Spinae Plane Block (ESPB), alongside techniques that specifically target visceral pain. Additionally, strategies for enhancing block efficacy through pharmacological adjuncts will be explored, providing a comprehensive guide to delivering precise and personalized analgesia for patients undergoing laparoscopic gynecological surgery.

### 1.1 Literature search strategy

This narrative review was conducted in accordance with the SANRA (Scale for the Assessment of Narrative Review Articles) guidelines (10). A comprehensive literature search was performed using PubMed and Web of Science. The search covered publications from database inception through January 2000 to October 2025. Search terms included combinations of: ‘laparoscopic gynecological surgery,’ ‘regional nerve block,’ ‘transversus abdominis plane block,’ ‘quadratus lumborum block,’ ‘erector spinae plane block,’ ‘postoperative analgesia,’ ‘Enhanced Recovery After Surgery,’ and ‘fascial plane block.’ Additional relevant studies were identified through manual screening of reference lists of included articles and existing systematic reviews. We prioritized randomized controlled trials, systematic reviews/meta-analyses, and Cochrane reviews, while also including observational studies, cadaveric studies, and case series where higher-level evidence was limited. No formal risk of bias assessment was conducted, consistent with the narrative nature of this review.

## 2 The genesis of regional techniques: foundational concepts and a technological leap

The evolution of pain management in laparoscopic gynecological surgery has progressed from basic techniques to sophisticated interventions, driven by advancements in technology.

### 2.1 Early attempts at targeted analgesia: infiltration and instillation

One of the earliest strategies for managing laparoscopic pain involved the intraperitoneal instillation of local anesthetics. Chou and colleagues demonstrated that this method effectively reduced visceral pain but had no impact on somatic or referred shoulder-tip pain (11). Similarly, continuous postoperative intraperitoneal nebulization of ropivacaine was investigated, but studies showed it neither improved pain control nor reduced analgesic consumption (12). Another approach aimed at somatic pain involved the direct infiltration of local anesthetics into trocar sites. Alessandri et al. (13) reported that presurgical infiltration with levobupivacaine reduced postsurgical wound pain and decreased rescue analgesic requirements, though the benefits were largely confined to the first 12 postoperative hours.

These early techniques underscored the limitations of targeting either somatic or visceral pain in isolation. They highlighted the multifactorial nature of post-laparoscopic pain and the need for more comprehensive approaches that address all pain components.

### 2.2 The ultrasound revolution: a new gold standard

The true transformation in regional anesthesia occurred with the clinical adoption of ultrasound guidance. Prior to this, peripheral nerve blocks relied on landmark-based techniques or nerve stimulation, which were inherently imprecise and carried higher risks of complications (14). The integration of ultrasound imaging into anesthetic practice revolutionized the field (15). Ultrasound allowed anesthesiologists to directly visualize target structures and surrounding anatomy, enabling precise needle placement and real-time observation of local anesthetic spread. This innovation resulted in higher success rates, faster onset times, reduced local anesthetic volumes, and improved safety profiles (15, 16).

Ultrasound guidance quickly became recognized as the new benchmark for regional anesthesia. Hopkins described it as the “gold standard,” a position that gained widespread acceptance (17, 18). The ability to visualize anatomical structures represented a transformative leap forward in both safety and efficacy.

Most importantly, ultrasound guidance unlocked the potential of fascial plane blocks, enabling the development and widespread adoption of advanced truncal block techniques such as the TAPB and QLB. These techniques have become increasingly important components of modern multimodal analgesia, though the strength of evidence varies across specific surgical contexts.

## 3 The workhorses: a comparative analysis of abdominal wall blocks

Ultrasound-guided fascial plane blocks targeting the nerves innervating the abdominal wall have become indispensable components of multimodal analgesia for laparoscopic gynecological surgery. These blocks provide targeted somatic analgesia while significantly reducing opioid requirements.

### 3.1 TAPB

#### 3.1.1 Anatomical basis, mechanism, and evolution of approaches

The TAPB targets the anterior rami of thoracolumbar spinal nerves (T7-L1) by depositing local anesthetic between the internal oblique and transversus abdominis muscles. These nerves supply sensory innervation to the abdominal wall skin, muscles, and parietal peritoneum (19). Initially performed using a landmark-based approach, the TAPB has evolved into an ultrasound-guided procedure, enabling precise anesthetic placement. This evolution has further led to the development of posterior, lateral, and oblique subcostal approaches (20).

Carney et al. (21) demonstrated that posterior injections produce more extensive and cranial spread compared to anterior injections, potentially reaching the paravertebral space. Anatomical studies indicate that TAPB injections administered cephalad to the iliac crest primarily involve T10-L1 nerve roots, making this technique particularly suited for lower abdominal surgeries (22).

#### 3.1.2 Evidence and efficacy in laparoscopic gynecological surgery

A Cochrane review by Charlton et al. (19) found that TAPB effectively reduces opioid consumption and pain scores following abdominal surgery. In laparoscopic gynecological surgery, several studies reported benefits. Sethi and Garg (23) and Ranjit and Shrestha (24) demonstrated that ultrasound-guided TAPB provided superior analgesia compared to local wound infiltration, with similar advantages observed in robotic-assisted procedures (25). However, the evidence is not universally favorable. Ghisi et al. (26) reported that bilateral TAPB did not significantly reduce 24-h morphine consumption or improve pain scores in patients undergoing total laparoscopic hysterectomy. A Cochrane review by Alsamman et al. (27), analyzing 21 trials, concluded that TAPB likely results in little to no clinically meaningful difference in postoperative pain or opioid consumption for minimally invasive gynecological procedures.

The heterogeneity in reported outcomes for TAPB in laparoscopic gynecological surgery can be attributed to several methodological and clinical factors. First, the various TAPB approaches (lateral, posterior, oblique subcostal) produce differing extents and patterns of local anesthetic spread, with posterior approaches demonstrating more cranial spread compared to anterior ones (21). Second, the timing of block administration relative to surgery (pre-incisional vs. post-surgical) varies considerably across studies. Third, heterogeneity in baseline multimodal analgesic protocols confounds comparisons, as robust non-opioid regimens may diminish the incremental benefit of any single intervention. Fourth, inconsistencies in outcome measures including different pain assessment tools, time points for evaluation, and definitions of opioid consumption limit direct comparison. Finally, the Cochrane review by Alsamman et al. (27) specifically noted that many included trials were at moderate-to-high risk of bias due to small sample sizes and inadequate blinding. These factors collectively explain why TAPB may appear to offer little clinical benefit in some analyses while showing clear advantages in others, rather than reflecting a true absence of efficacy.

### 3.2 QLB

The limitations of the TAPB prompted the exploration of the QLB, which potentially provides more comprehensive analgesia by addressing both somatic and visceral pain components.

#### 3.2.1 Anatomical concepts and proposed mechanisms for broader analgesic coverage

The QLB targets the fascial planes surrounding the quadratus lumborum muscle. Unlike the TAPB, local anesthetic deposited around the quadratus lumborum muscle can migrate medially toward the paravertebral space via the multi-layered thoracolumbar fascia (28). This paravertebral spread may block sympathetic fibers from the thoracic sympathetic chain or afferent visceral fibers traveling with the splanchnic nerves (28, 29).

Adhikary et al. (30) demonstrated via imaging that a transmuscular QLB results in injectate spread to the lumbar paravertebral space, suggesting a mechanism similar to that of a lumbar plexus block. Multiple approaches to QLB have been described, including lateral (QL1), posterior (QL2), and anterior/transmuscular (QL3), each with distinct spread patterns and clinical effects (31, 32).

#### 3.2.2 Comparative efficacy in gynecological surgery: evidence and debate

Clinical evidence suggests that QLB may provide superior analgesia compared to TAPB in certain surgical contexts. Murouchi et al. (33) found that posterior QLB provided significantly longer analgesia following laparoscopic ovarian cystectomy as compared to lateral TAPB. Similarly, Aoyama et al. (34) observed comparable effects between QL2 and posterior TAPB, while Ishio et al. (35) demonstrated significantly lower pain scores for up to 24 h with posterior QLB compared to controls.

In direct comparisons, Huang et al. (36) found that transmuscular QLB significantly reduced morphine consumption and visceral pain scores compared to oblique subcostal TAPB in laparoscopic hysterectomy. Baran (37) also demonstrated that both anterior QLB and ESPB effectively reduced opioid consumption following total laparoscopic hysterectomy.

However, not all studies favor QLB. Elfeky et al. (38) found no significant difference between QLB and control groups in patients undergoing minimally invasive hysterectomy. Additionally, Kim et al. (39) reported that rectus sheath block was superior to QLB in single-port adnexal surgery. Despite promising findings, several important limitations must be acknowledged. The QLB is a technically more demanding procedure compared to TAPB, particularly the anterior/transmuscular approach, which requires visualization of deeper anatomical structures and longer needle pathways. This raises concerns about operator dependency, a factor that is rarely controlled for in existing RCTs. The multiple described approaches (QL1, QL2, QL3) with distinct spread patterns and potentially different clinical effects introduce further heterogeneity, and the optimal approach for gynecological surgery remains undetermined. Additionally, the proposed mechanism of paravertebral spread, while supported by imaging studies (30), is not consistently demonstrated in all cadaveric or clinical investigations, and the degree to which visceral analgesia is truly achieved via this mechanism remains debated.

Safety considerations for QLB include the risk of local anesthetic systemic toxicity, particularly with bilateral blocks requiring larger total volumes of local anesthetic. Lower limb motor weakness is a recognized complication, especially with the anterior (transmuscular) approach, due to potential spread to the lumbar plexus. Femoral nerve weakness has been reported, potentially delaying mobilization, a key component of ERAS pathways. Transient hypotension from sympathetic blockade has also been described. The learning curve for QLB, particularly the transmuscular approach, is steeper than for TAPB, requiring proficiency in deep sonographic visualization and needle manipulation with curvilinear probes.

The following table summarizes the key comparative features of the TAPB and QLB based on the available literature (Table 1).

**Table 1:** The key comparative features of the TAPB and QLB.

Feature	TAPB	QLB
Primary target	Nerves within the fascial plane between internal oblique and transversus abdominis muscles	Nerves within fascial planes surrounding the quadratus lumborum muscle
Analgesic coverage	Primarily somatic (anterolateral abdominal wall)	Somatic and visceral (potential spread to paravertebral space)
Innervation blocked	Primarily thoracolumbar nerves (T7-L1)	Thoracolumbar nerves (T6-L3) and potential lumbar plexus/sympathetic involvement
Efficacy for visceral pain	Limited to poor (11, 26)	Moderate to good (35, 36)
Duration of analgesia	Typically 8–12 h (32)	Often extends beyond 24 h (32, 33)
Key clinical evidence	Recent Cochrane review suggests minimal clinical benefit (26, 27)	Multiple RCTs show superiority over TAPB for visceral pain and opioid sparing (33, 36)
Potential complications	Visceral injury (blind technique), local anesthetic systemic toxicity	local anesthetic systemic toxicity, lower limb motor weakness (especially with anterior approach) (76), hypotension

### 3.3 ESPB

The ESPB has emerged as a technically simpler and potentially safer alternative to deeper blocks, offering advantages in ease of execution and reduced risk of complications.

#### 3.3.1 Technique and postulated widespread analgesic effect

The ESPB involves the injection of local anesthetic deep to the erector spinae muscle and superficial to the vertebral transverse processes. Its mechanism of action is postulated to include extensive cranio-caudal spread within the fascial plane and anterior spread through the costo-transverse foramina into the paravertebral and epidural spaces (40). This allows the block to influence both somatic and visceral components of pain by affecting spinal nerve rami and the sympathetic chain.

#### 3.3.2 Emerging evidence and clinical application

Evidence supporting ESPB in laparoscopic gynecological surgery is growing. Frassanito et al. (40) reported that bilateral ESPB at the T10 level resulted in low postoperative pain scores and minimal morphine consumption following total laparoscopic hysterectomy. Similarly, Baran (37) demonstrated that both ESPB and QLB were equally effective in reducing postoperative tramadol consumption and pain scores after hysterectomy, with ESPB offering the added advantage of shorter performance time. A meta-analysis by Qian et al. (41) further reinforced the efficacy of ESPB, concluding that it was superior to TAPB for postoperative analgesia and opioid reduction across various abdominal surgeries.

Despite its growing popularity, several important limitations of ESPB warrant discussion. The mechanism of action of ESPB remains a subject of active scientific debate. While anterior spread through the costo-transverse foramina into the paravertebral space has been postulated, cadaveric and imaging studies have shown inconsistent results regarding the reliability and extent of this spread.

The ESPB is an emerging regional anesthetic technique with significant potential for clinical benefit, yet its exact mechanism(s) of action has been much debated (42). The ESPB has an uncertain mechanism of action with unpredictable sensory and motor blocking profiles that further limit its suitability as the ultimate Plan A block (43). A volume of 20 mL of local anesthetic appears to result in cutaneous blockade of as few as zero and as many as 13 dermatomal segments, with no obvious technical reason for the disparity (44). Furthermore, numerous studies have shown that the ESPB does not spread to the paravertebral space in a reliable and reproducible way (45). An alternative hypothesis, proposed by Lönnqvist et al. (45), suggests that the analgesic effect may partly be mediated through systemic absorption of local anesthetic rather than a traditional nerve-blocking mechanism. These ongoing debates underscore that the evidence base, while encouraging, does not yet support definitive claims of broad clinical superiority for ESPB.

Although ESPB is generally considered to have a favorable safety profile given its superficial injection site, complications are not negligible.” Infection at the needle insertion site, local anesthetic toxicity/allergy, vascular puncture, pleural puncture, pneumothorax, and failed block are the primary complications. Anecdotal complications have been described in patients undergoing ESP block, such as pneumothorax and motor block, and recent evidence demonstrated a rapid absorption from the ESP plane, possibly leading to local anesthetic systemic toxicity (46). Given that the true incidence of major complications after ESPB is likely to be low, more large studies reporting complications and efficacy are required before concluding safety benefits of ESPB compared to other regional techniques (47). The block failure rate also warrants discussion, as the inconsistency in analgesic spread may result in inadequate analgesia in a proportion of patients.

While ESPB is frequently described as ‘technically simple,’ this characterization primarily applies to the needle insertion technique itself. Accurate sonographic identification of the transverse process and correct fascial plane deposition require supervised training and experience. Similarly, all QLB approaches require competence in deep ultrasound-guided techniques, and performance variability between operators is a recognized confounding factor in clinical studies.

### 3.4 Other relevant truncal blocks

Several other truncal blocks have more specialized roles, tailored to specific incision patterns or used as adjuncts in multimodal analgesia strategies.

#### 3.4.1 Rectus sheath block (RSB)

The RSB provides targeted analgesia for midline and periumbilical incisions by blocking the anterior cutaneous branches of thoracolumbar nerves (T9-T11) (48). Cho et al. (48) demonstrated that RSB significantly reduced pain scores and rescue analgesic requirements in patients undergoing laparoscopic gynecological surgery. Further refining its utility, Siripruekpong et al. (49) explored the minimal effective dose of anesthetic for ambulatory procedures, highlighting the block’s role in facilitating early discharge. In comparative studies, Kim et al. (39) reported that RSB was superior to QLB for single-port adnexal surgery. However, its coverage is restricted to the medial abdominal wall, limiting its application to localized midline pain.

#### 3.4.2 Llioinguinal/Iliohypogastric (II/IH) nerve block

The Ilioinguinal/Iliohypogastric (II/IH) nerve block targets the L1 nerve roots, providing sensory blockade to the iliac crest, groin, and suprapubic region. Yucel et al. (50) demonstrated that the II/IH block enhanced analgesia following total abdominal hysterectomy. However, its utility in modern laparoscopic gynecological surgery has diminished. These procedures often require broader analgesic coverage than the II/IH block provides, leading to its replacement by more extensive fascial plane blocks like TAPB, QLB, or ESPB.

## 4 Targeting the core: advanced blocks for visceral pain control

A substantial portion of postoperative discomfort following laparoscopic gynecological surgery is attributed to visceral pain, primarily transmitted via the autonomic nervous system. Such pain is often inadequately addressed by truncal blocks. This chapter explores regional techniques specifically designed to intercept visceral pain signals, with a particular focus on the Superior Hypogastric Plexus Block (SHPB) and neuraxial anesthesia.

### 4.1 SHPB

The SHPB is a targeted intervention for pelvic visceral nociception, achieved by blocking the superior hypogastric plexus. This retroperitoneal network of autonomic nerves is situated anterior to the L5 vertebra and the sacral promontory. The plexus conveys afferent pain fibers from pelvic organs such as the uterus, cervix, and proximal fallopian tubes.

Rapp et al. (51), in a double-blind randomized clinical trial, demonstrated that injecting ropivacaine into the presacral space during open abdominal hysterectomy significantly reduced postoperative opioid consumption and pain scores compared to placebo. Systematic reviews by Shama et al. (52) and Salem et al. (53) corroborated these findings, confirming that SHPB effectively reduces pain scores, opioid consumption, and Postoperative Nausea and Vomiting (PONV) in hysterectomy patients.

However, the SHPB’s role in laparoscopic gynecological surgery remains nuanced, with benefits appearing less pronounced than in open surgery. This reduced effect may stem from the lower intensity of visceral pain in laparoscopic procedures, widespread use of multimodal ERAS protocols, and technical challenges of laparoscopic block performance.

### 4.2 The role of neuraxial anesthesia

Neuraxial anesthesia offers profound analgesia for abdominal and pelvic surgeries by concurrently blocking somatic and visceral dermatomes. Studies, such as those by Gerges et al. (1), highlight its potential advantages for laparoscopy, including faster recovery and reduced PONV. Applying neuraxial techniques to laparoscopic surgery presents unique challenges, primarily sympathetic blockade leading to hypotension (exacerbated by pneumoperitoneum) and potential motor blockade delaying ambulation-a key ERAS component.

To address these challenges, modern approaches increasingly favor lower-dose, combined techniques that preserve analgesic efficacy while minimizing complications. Zdravkovic and Kamenik (54) demonstrated that combining general anesthesia with low-dose spinal anesthesia significantly reduced both intraoperative and postoperative opioid consumption without compromising hemodynamic stability. While Seki et al. (55) reported that epidural anesthesia improved patient satisfaction through its opioid-sparing effects.

Neuraxial anesthesia, therefore, remains a valuable tool not as a standalone technique but as a flexible component of individualized anesthetic plans. It is particularly beneficial for complex surgeries or patients requiring superior pain control. Together with SHPB, these advanced techniques enhance the anesthesiologist’s ability to effectively manage visceral pain in gynecological procedures.

## 5 The pursuit of perfection: strategies for optimizing block efficacy and duration

The limited duration of fascial plane blocks continues to pose a significant challenge in perioperative pain management. To address this limitation, clinical research has concentrated on three primary strategies: augmenting local anesthetics with pharmacological adjuvants, optimizing local anesthetic dosing, and developing novel, long-acting formulations.

### 5.1 Pharmacological adjuncts: extending and enhancing the block

The addition of pharmacological adjuvants to local anesthetic solutions offers a promising approach to extend the duration and improve the quality of single-injection regional blocks. A systematic review by Kirksey et al. (56) provided a comprehensive overview of these agents.

Dexamethasone has emerged as one of the most effective adjuvants. Zhang et al. (57) demonstrated that dexamethasone significantly prolonged analgesia, reduced opioid consumption, and lowered PONV incidence in TAPB. While its precise mechanism remains debated, evidence suggests that both perineural and intravenous administration provide comparable benefits (58).

Dexmedetomidine, an alpha-2 adrenergic agonist, enhances analgesia by inducing localized vasoconstriction and inhibiting nerve fiber action potentials. However, its use must be carefully managed due to potential side effects, including sedation and bradycardia (59, 60).

Magnesium sulfate acts by modulating nociceptive signaling through NMDA receptor and calcium channel blockade. Studies have shown that magnesium sulfate prolongs analgesia and reduces morphine consumption in TAPB and QLB (61, 62). Perineural opioids, while effective, have been limited in their use due to adverse effects, rendering them less favorable in the context of opioid-sparing strategies (56).

### 5.2 Dose optimization: finding the sweet spot

Optimizing the dose, concentration, and volume of local anesthetics seeks to balance effective analgesia with minimal drug exposure, thereby reducing the risk of toxicity. This approach has shifted clinical practice from empirical dosing to evidence-based strategies. Wang et al. (63) employed sequential allocation methods to determine the optimal concentration of ropivacaine for QLB, identifying a dosing strategy that maximizes effectiveness while minimizing adverse effects. Similarly, Siripruekpong et al. (49) found that lower doses of bupivacaine were sufficient for RSB when used as part of multimodal analgesic pathways. This transition to dose finding studies underscores the importance of precise dosing strategies tailored to individual patient needs and surgical contexts.

### 5.3 Advances in local anesthetics

The development of novel, long-acting formulations of local anesthetics represents the third approach to optimizing block efficacy and duration. Liposomal bupivacaine, an extended-release formulation encapsulated within multivesicular liposomes, allows for sustained release of the drug over 72 h following a single administration (64, 65).

While some meta-analyses have demonstrated its effectiveness in reducing pain and opioid consumption, its clinical superiority over conventional formulations remains under scrutiny. Although the direct drug cost of liposomal bupivacaine is substantially higher than that of conventional formulations, emerging pharmacoeconomic evidence suggests that it may prove cost-effective at the health-system level. Studies in colorectal and orthopedic surgery have demonstrated that the use of liposomal bupivacaine can reduce total cost of care by decreasing the need for additional analgesics, lowering complication rates such as postoperative ileus, and shortening hospital length of stay (66, 67). However, it should be noted that dedicated cost-effectiveness analyses specific to fascial plane blocks in gynecological surgery are lacking, and generalizing economic findings across surgical specialties warrants caution. Further comparative trials are required to clarify its role in fascial plane blocks, particularly in laparoscopic gynecological surgeries, where both analgesic and economic benefits remain to be definitively established (Table 2).

**Table 2:** Summary of key RCTs evaluating liposomal bupivacaine in fascial plane blocks.

Study	Design	Surgery type	Block type	Main findings
Hutchins et al. (77)	Prospective, RCT, observer-blinded	Robotic-assisted hysterectomy	Subcostal TAPB (LB vs. bupivacaine)	LB significantly reduced opioid consumption at 72 h and lowered maximal pain scores
Hutchins et al. (78)	Prospective, RCT, double-blinded	Robotic/laparoscopic hysterectomy	Subcostal TAPB (LB) vs. port-site infiltration (bupivacaine)	LB TAPB reduced 72 h opioid consumption and improved quality of recovery
Turan et al. (79)	Multicenter, RCT	Major abdominal surgery	TAPB (LB) vs. continuous epidural analgesia	LB TAPB noninferior for 72 h pain scores; lower hypotension rates; higher opioid consumption
Antony et al. (80).	Pilot, RCT, single-blinded	Cesarean delivery	Surgical TAPB (LB + bupivacaine vs. bupivacaine alone)	No significant difference in 48 h MME; trend toward reduced opioid use with LB
Marciniak et al. (81)	RCT	Minimally invasive thoracic surgery	PECS + SAPB (LB + bupivacaine vs. bupivacaine alone)	LB did not improve OBAS, reduce opioid use, or decrease pain scores
Hussain et al. (82)	Systematic review and meta-analysis of RCTs	Abdominal surgery (pooled)	Various abdominal FPBs (LB vs. plain LA)	Similar analgesic effectiveness between LB and plain LA for abdominal fascial plane blocks

LB, liposomal bupivacaine; TAPB, transversus abdominis plane block; PECS, pectoralis nerve block; SAPB, serratus anterior plane block; FPB, fascial plane block; LA, local anesthetics; MME, morphine milligram equivalents; OBAS, overall benefit of analgesia score.

## 6 Synthesis and future horizons

The evolution of regional nerve blocks in laparoscopic gynecological surgery exemplifies a broader shift toward patient-centered, evidence-based pain management. This section synthesizes key findings and explores future directions for advancing regional anesthesia in this field.

### 6.1 Integration into practice: the block as a pillar of ERAS

Regional nerve blocks have become a cornerstone of ERAS protocols, playing a crucial role in minimizing surgical stress and expediting functional recovery.

One of the most significant contributions of regional blocks is their opioid-sparing effect. A network meta-analysis by Ding et al. (8) demonstrated that combining regional techniques with non-opioid analgesics significantly reduces perioperative opioid consumption. This reduction is critical in mitigating opioid-related side effects, such as nausea, sedation, and ileus, which can delay recovery and prolong hospital stays (68).

Effective regional analgesia also facilitates quicker functional recovery, with evidence showing that multimodal protocols incorporating regional anesthesia promote earlier ambulation and faster return of bowel function (7). Beyond physical recovery, regional anesthesia enhances psychological well-being, yielding higher scores in quality-of-recovery assessments across domains such as physical comfort, emotional state, and pain relief (69).

### 6.2 Toward personalized analgesia: which block for which surgery?

The clinical focus in regional anesthesia has shifted from determining whether a block should be used to identifying the most appropriate block for a given procedure and patient. This approach aims to tailor analgesic strategies to the unique “pain fingerprint” of each surgery.

The concept of the surgical ‘pain fingerprint’ can be operationalized by considering three dimensions: (1) the relative contribution of somatic versus visceral pain, which is largely determined by the surgical procedure; (2) the number and location of trocar sites, which determines the required dermatomal coverage; and (3) the anticipated duration and severity of pain, which informs the choice between single-shot blocks, adjuvant-enhanced blocks, or catheter-based techniques. For procedures with a predominantly visceral pain component, such as total laparoscopic hysterectomy, blocks with potential paravertebral spread QLB or ESPB are preferred over TAPB. Conversely, for single-port procedures with localized somatic pain, a targeted RSB may be more appropriate than a broader fascial plane block. This framework should be considered a starting point for clinical decision-making rather than a definitive algorithm, as high-quality head-to-head comparative data for many procedure-block combinations remain limited.

The choice of block depends on the nature of the procedure. For total laparoscopic hysterectomy, which involves significant visceral and back pain, regional techniques with visceral analgesic capabilities, such as the QLB or ESPB, have demonstrated efficacy (4, 36, 37). Conversely, procedures characterized by predominantly localized somatic pain, such as minor laparoscopic interventions, may benefit more from a RSB (39).

Post-laparoscopic pain is inherently multifactorial, with components such as referred shoulder-tip pain often escaping the coverage of abdominal wall blocks. Therefore, a comprehensive strategy that combines regional techniques with interventions targeting specific pain mechanisms is essential (70). The proposed clinical framework is outlined in Table 3.

**Table 3:** Proposed clinical framework: matching regional block selection to laparoscopic gynecological procedure type.

Procedure	Dominant pain components	Suggested primary regional technique(s)	Adjunctive considerations	Level of evidence
Total laparoscopic hysterectomy	Visceral (predominant) + Somatic + Back pain	QLB (transmuscular/posterior) or ESPB	Consider SHPB for refractory visceral pain; pharmacological adjuvants for prolonged effect	Moderate (multiple RCTs)
Laparoscopic ovarian cystectomy	Somatic (moderate) + Visceral (mild–moderate)	QLB (posterior) or TAPB (posterior)	Shorter duration procedure; TAPB may suffice for simpler cases	Moderate
Single-port laparoscopic adnexal surgery	Somatic (localized, periumbilical)	RSB	Limited trocar sites favor midline-targeted blocks	Low-moderate (limited head-to-head data)
Diagnostic laparoscopy / Minor procedures	Somatic (mild, port-site)	TAPB (lateral) or Local wound infiltration	Block may offer marginal benefit over robust multimodal analgesia alone	Low (Cochrane review suggests limited benefit)
Laparoscopic deep endometriosis surgery	Visceral (significant) + Somatic + Neuropathic	QLB or ESPB ± SHPB	Complex pain profile; may benefit from neuraxial adjunct in selected cases	Very low (extrapolated)
Robotic-assisted gynecological surgery	Somatic + Visceral (variable by procedure)	TAPB or QLB (depending on scope of surgery)	Port placement patterns may vary; block selection should match incision distribution	Low-moderate

Recommendations are based on available evidence and clinical rationale; many comparisons lack direct head-to-head RCTs in the specific gynecological context. Individual patient factors (comorbidities, anticoagulation status, body habitus, surgeon preference) should also guide block selection. All regional techniques should be integrated within a multimodal analgesic strategy.

### 6.3 The next frontier: the influence of technology and innovation

The future of regional anesthesia is being shaped by technological innovation, which promises to refine existing techniques and introduce groundbreaking advancements. The following discussion distinguishes between technologies with existing evidence base and those that remain at early stages of development. Readers should interpret the latter as identifying potential avenues of investigation rather than imminent clinical practice changes.

Refined approaches, such as the modified subcostal anterior QL block, have demonstrated superior opioid-sparing effects when compared to traditional methods (71). Algorithms capable of identifying anatomical structures and guiding real-time needle placement during ultrasound-guided blocks are under development (72). These advancements could democratize regional anesthesia by shortening learning curves, standardizing performance, and improving procedural safety.

Innovations in pharmacology are driving the development of ultra-long-acting local anesthetics, capable of extending the duration of single-shot blocks to 72 h or longer. The integration of robotics into regional anesthesia offers the potential for superhuman precision in needle placement and drug delivery. Further innovations include ultra-long-acting local anesthetics that extend the duration of single-shot blocks to 72 h and beyond, and the integration of robotics capable of superhuman precision in needle placement.

Artificial Intelligence (AI) represents a transformative development in regional anesthesia. The application of AI to ultrasound-guided regional anesthesia has progressed beyond the conceptual stage, with several published studies demonstrating feasibility. AI for ultrasound scanning in regional anaesthesia is a rapidly developing interdisciplinary field, though there is a risk that work could be undertaken in parallel by different elements of the community with a lack of knowledge transfer between disciplines (72). AI technology in this domain has achieved an accuracy of 99.7% in identifying specific anatomical structures in some evaluations (73). The Guidance for Reporting Artificial Intelligence Technology Evaluations for Ultrasound Scanning in Regional Anesthesia (GRAITE-USRA) guideline, published in 2025, is the first international reporting framework developed for the scientific evaluation of AI applications in ultrasound-guided regional anesthesia (74).

However, it is important to acknowledge the limitations of current AI technology. Most published studies evaluate AI performance in optimal conditions, and real-world clinical validation remains limited. The future of regional anesthesia with AI integration appears promising, yet obstacles such as device malfunction, data privacy, regulatory barriers, and cost concerns can deter its clinical implementation (75).

Robotic-assisted needle placement in regional anesthesia remains at a pre-clinical development stage, and its clinical utility has not yet been demonstrated in randomized studies. Similarly, while ultra-long-acting formulations such as liposomal bupivacaine have been studied in various surgical contexts, their cost-effectiveness and specific role in fascial plane blocks for laparoscopic gynecological surgery await clarification in well-designed comparative trials. These technologies represent areas of active investigation rather than near-term clinical solutions.

## 7 Conclusion

Regional anesthesia for laparoscopic gynecological surgery has advanced from basic techniques to highly precise and personalized approaches. These blocks now serve as fundamental pillars of perioperative care and integral elements of ERAS protocols, offering effective opioid-sparing analgesia and facilitating improved postoperative recovery. The current paradigm emphasizes tailoring anesthesia strategies to the specific needs of both the procedure and the individual patient.
